# Using Dairy Value Chains to Identify Production Constraints and Biosecurity Risks

**DOI:** 10.3390/ani10122332

**Published:** 2020-12-08

**Authors:** Jaswinder Singh, Balbir B. Singh, Harish Kumar Tiwari, Harmandeep Singh Josan, Nidhi Jaswal, Manmeet Kaur, Polychronis Kostoulas, Mehar Singh Khatkar, Rabinder Singh Aulakh, Jatinder Paul Singh Gill, Navneet K. Dhand

**Affiliations:** 1 Department of Veterinary &Animal Husbandry Extension Education, Guru Angad Dev Veterinary and Animal Sciences University, Ludhiana 141004, India; jaswindervet97@gmail.com (J.S.); josanharmanvet@gmail.com (H.S.J.); 2 School of Public Health and Zoonoses, Guru Angad Dev Veterinary and Animal Sciences University, Ludhiana 141004, India; bbsdhaliwal@gmail.com (B.B.S.); rsaulakh@gmail.com (R.S.A.); gilljps@gmail.com (J.P.S.G.); 3 Sydney School of Veterinary Science, The University of Sydney, Camden 2570, New South Wales, Australia; harish.tiwari@sydney.edu.au (H.K.T.); mehar.khatkar@sydney.edu.au (M.S.K.); 4 School of Public Health, Postgraduate Institute of Medical Education and Research, Chandigarh 160012, India; nidhi.jaswal@gmail.com (N.J.); mini.manmeet@gmail.com (M.K.); 5 Faculty of Veterinary Medicine, University of Thessaly, 43100 Karditsa, Greece; pkost@vet.uth.gr

**Keywords:** dairy value chain, farm biosecurity, cattle infectious diseases, value chain mapping, risk nodes

## Abstract

**Simple Summary:**

The dairy industry plays a key role in the Indian economy. This study was conducted to understand the dairy inputs and outputs and to identify production constraints and biosecurity. Focus group discussions and key informant interviews were conducted in the Punjab state of India with 119 participants, including veterinarians, paraveterinarians, veterinary academics, dairy farmers and key informants. Farm inputs (e.g., feed and animal health services) and outputs (e.g., milk, animal sales, carcass and manure disposal) were mapped. Production constraints and biosecurity practices were identified and included the availability of green and dry fodder, provision of veterinary services by untrained practitioners, improper disposal of carcass/placenta/excreta, absence of health certification during sale or purchase of animals and absence of testing of village bulls. The government was a major provider of health and management services in the state, although a very high proportion of farmers relied on untrained or partially trained service providers for health advice and veterinary procedures. Improvement in biosecurity practices and adequate use of personal protective equipment is recommended to reduce the incidence of infectious diseases and minimize their impact.

**Abstract:**

The dairy industry plays an important role in the economy and food security of India. A study of the dairy value chains was conducted in Punjab, India, to identify production constraints and biosecurity risks. Focus group discussions and key informant interviews were conducted during 2018–2019 with a total of 119 participants comprising veterinarians (41), paraveterinarians (15), veterinary academics (12), dairy farmers (46) and key informants (5). Input and output value chains were created, and potential risk nodes were identified that could facilitate the transmission of pathogens between animals, farms and villages. The majority of the participants were male (93%), middle-aged (68%) or worked in rural areas (75%). Most of the farmers self-cultivated their green fodder (82%), used the wheat straw from their own fields (60%) but purchased commercial feed (63%). Artificial insemination was used by 85% of farmers for cattle, but only 68% for buffaloes. Most of the farmers (76%) reported getting their animals vaccinated against foot-and-mouth disease and hemorrhagic septicemia. Animals were sold and purchased without any health certification and testing in most cases. Adoption of biosecurity measures by farmers and the use of personal protective equipment by veterinary personnel were very low. We recommend conducting epidemiological studies to further characterize the identified risk nodes, training of veterinary practitioners and farmers to ensure adequate biosecurity practices and the appropriate use of personal protective equipment.

## 1. Introduction

The dairy industry has emerged as a key player in the Indian economy. Dairying not only serves as a major source of income for rural India, but also provides employment for small scale and marginal farmers, empowers women and generates foreign exchange [1,2]. India has the largest dairy animal population in the world (302 million), producing 187.7 million tons of milk in 2019 [3]. Although the per capita availability of milk (394 g/day) in India is higher than the world average of 294.2 g/day, it falls short of recommended international standards for average nutritional requirements vis-à-vis milk consumption [1,3]. The growth of the dairy industry in India is essentially market driven due to an increased demand for milk and milk products from the rising middle-income class. This demand is propelling the industry to grow at a rate of 6.5%, which is nearly double the growth rate of the agriculture sector (2.7%) [2,4]. Nonetheless, India’s potential to further increase dairy production and increase the share of its exports largely depends on enhancing the productivity of the milking herd. This would require changes to its small-scale dairy husbandry structure along with improved animal feeding practices and disease control [4]. It is imperative to understand the input costs, drivers of productivity, actors involved with product safety and quality and the factors that reduce the incidence of diseases in the dairy herds to achieve such potential. Further, it is important to assess the structure of the existing animal health services, along with their strengths and constraints. It is also vital to understand how different actors in the industry interact and how outputs from the dairy farm are sold or marketed before developing any recommendations to improve the system.

Value chains describe the process or activities involved in procuring, producing and marketing of products. The exploration of value chains is an effective tool that has been frequently used in India and elsewhere to identify the constraints related to improving the efficiency and scale of dairy production. It also helps in mitigating the market risks [5]. Ideally, a value chain analysis should be a critical evaluation of the chains involved, and the actors involved in various components of the industry, and their inter-relationships [6,7]. It provides information about the flows of inputs to the production system and outputs from the system, thus providing a holistic view of the industry [7]. Value chains also allow the identification of weak points or “risk nodes” in the system that make the system potentially vulnerable to diseases or other hazards. Identification of such nodes can give insights into approaches that could be used to control diseases [7]. Value chain mapping has been successfully used to understand various livestock production systems and to identify risk nodes for disease spread in several countries including Ethiopia, the United States, Kenya and Sri Lanka [8,9,10,11]. However, studies of the value chains in the dairy sector in India are focused primarily on the institutionalized advancements in the milk marketing system and often do not explore the concerns regarding livestock procurement, health, nutrition and the veterinary care structure inputs.

Using value chains, Vamsidhar Reddy, et al. [12] identified limited transport and storage facilities, access and quality of health services and quality of artificial insemination services as important issues in the development of the dairy sector in rural India. Birthal, Chand, Joshi, Saxena, Rajkhowa, Khan, Khan and Chaudhary [5] assessed the performance and financing of dairy value chains. They reported that formal value chains led by dairy cooperatives and private processors, and informal value chains driven by vendors, local traders and consumer-households coexist in India. There are, however, few studies conducted in India that explore the input and output dairy value chains, and that identify the risks of disease transmission through the existing animal husbandry practices. The dairy value chain studies that simultaneously identify biosecurity risks, production constraints and challenges to the milk marketing networks are also few. Therefore, this study was conducted in the state of Punjab, India with the following objectives: (a) to study the existing dairy value chains, (b) to identify the gaps in the dairy value chains that hamper milk production and (c) to recognize risk nodes that facilitate the transmission of infections in animals and humans. The study identified production and biosecurity constraints including the availability of green and dry fodder, provision of veterinary services by untrained practitioners, improper disposal of carcass/placenta/excreta, absence of health certification during sale or purchase of animals and absence of testing of village bulls. A very high proportion of farmers relied on untrained or partially trained service providers for health advice and veterinary procedures.

## 2. Materials and Methods

Information about the input and output of the value chain was collected through a series of focus group discussions (FGDs) and key informant interviews (KIIs) followed by a structured questionnaire survey conducted at various locations in the state of Punjab, India. Three distinct groups: dairy farmers, veterinary academics and veterinary personnel (practicing field veterinarians and paraveterinarians) participated in the study. Paraveterinarians are paraprofessionals who hold a diploma in veterinary sciences and provide some veterinary services in animal health and management. The titles such as veterinary nurse, veterinary pharmacist, veterinary inspector, veterinary technician and veterinary assistant are used synonymously to address them.

### 2.1. Ethics Approval

The Human Ethical Research Committee, Dayanand Medical College and Hospital, Ludhiana provided the necessary approval to conduct this study (approval number DMCH/R&D/2018/273).

### 2.2. Study Area

The state of Punjab, India is located in the north west part of the country (31.1471° N, 75.3412° E) and has a total geographical area of 50,362 km^2^ [13]. The climate of the area is tropical, semiarid, hot and subtropical monsoon type with cold winters and hot summers, with an average annual temperature of 21 °C (up to 0 °C in winter to up to 47 °C in summer). About two-thirds of the population of the state lives in rural areas (62.5% of 27.7 million) [13]. Dairy farming is an integral part of state agricultural sector comprising approximately one-third of the total agriculture sector [14]. The rural population of the state is traditionally engaged in livestock production. However, most of the dairy farmers in the state are smallholders [15] and rear only one to four animals to generate income to supplement that from cropping, although few medium and large dairy farmers also participated in the study. Currently, Punjab is home to 69.92 million livestock including 2.47 million cattle and 4.01 million buffaloes [3]. Milk constitutes 80% of the total output from the livestock sector in the state, with Punjab currently producing 12.6 million tons of milk per year [3], 55% of which is sold as fluid milk [14].

### 2.3. Participants

Detailed methods are presented elsewhere [16]. Briefly, we invited government veterinarians and paraveterinarians from three subdistricts, one from each of the three regions (Majha, Malwa and Doaba) of the state to participate in FGDs between October 2018 and May 2019. Veterinary academics were selected from the Guru Angad Dev Veterinary and Animal Sciences University (GADVASU), the only veterinary university in the state. Dairy farmers were selected from three farmer training workshops organized by GADVASU. The KIIs were conducted with five stakeholders including two progressive dairy farmers, two milk vendors and one dairy product manufacturer. A progressive farmer is one who applies available knowledge and technology to improve the profitability of their farming enterprise. Milk vendors collect fresh milk from farmers and supply it to consumers. All participants (FGDs and KIIs) were invited to take the questionnaire survey subsequent to the discussions.

### 2.4. Data Collection

FGDs and KIIs were conducted in the Punjabi language.

At the outset, the participants were briefed about the study objectives and were then asked to identify various input and output chains. These were different sources of feed and fodder, sources of drinking water, sources and availability of semen for artificial insemination, provision of animal health services, use of biosecurity practices, level of use of personal protection, sale and purchase of animals, sale of milk and the disposal of carcass and effluents. The facilitator presented the identified value chains on a flipchart as the discussion progressed and asked the participants whether they agreed with the chain structure or if they had anything else to add. For the chains where multiple input or output sources were mentioned, participants were also asked to provide estimates of the respective inputs and outputs to identify the relative importance of value chains. The participants were encouraged by the facilitator to arrive at a consensus through discussion if there were variations in the values.

In addition to constructing value chains, the participants were also asked to identify potential risk points or vulnerabilities for the transmission of infectious diseases, with a particular focus on bovine brucellosis. When a node was identified as a risk point, they were asked to categorize the level of risk as low or high. The information about biosecurity practices and the use of personal protective equipment (PPE) was also obtained to identify transmission risk nodes. The FGDs were audio recorded. One of the coauthors (HS) also noted key discussion points.

Demographic data and information related to biosecurity measures, feed/fodder and animal health practices were collected from the participants using a self-completed questionnaire survey (available from the authors on request) subsequent to the FGDs and KIIs. Three separate custom-made questionnaires were used for veterinary practitioners, academics and farmers respectively.

### 2.5. Data Handling and Analysis

The written notes of FGDs were translated into English from Punjabi, and flip charts and audio recordings were reviewed. Chains of individual inputs and outputs were finalized and combined to develop a comprehensive value chain map of the existing dairy industry in the state. The dairy value chain maps were reviewed to ensure proper categorization.

Questionnaire data were transferred to Microsoft Excel (2007) for further analysis. Frequency tables were prepared for categorical variables and frequencies, and relative frequencies were calculated using SAS statistical program (release 9.4 © 2020 by SAS Institute Inc., Cary, NC, USA). Data were imported into the R programming environment for preparation of graphs [17] using the ‘R’ package “ggplot2” [18]. The FGD quotes were translated into English for presentation in this manuscript.

## 3. Results

**Figure 1:**
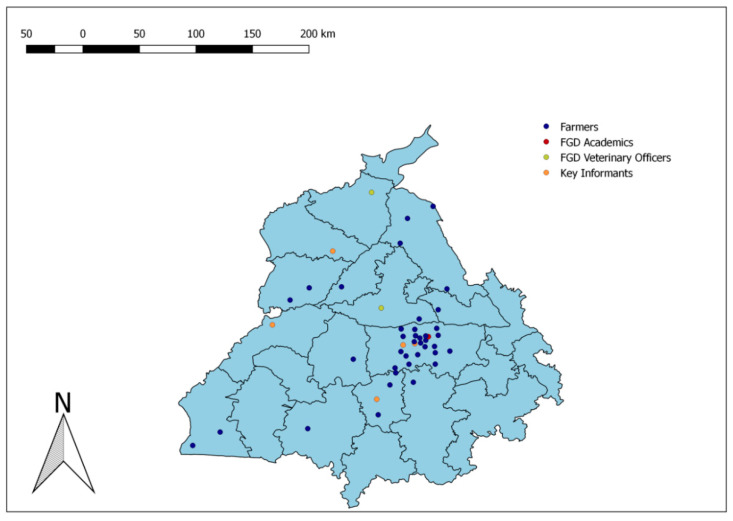
Locations of the residences and workplaces of farmers and veterinary practitioners who participated in the seven focus group discussions (FGDs) and five key informant interviews (KII) conducted during the study of the dairy value chains in Punjab state of India, 2018–2019.

**Figure 2:**
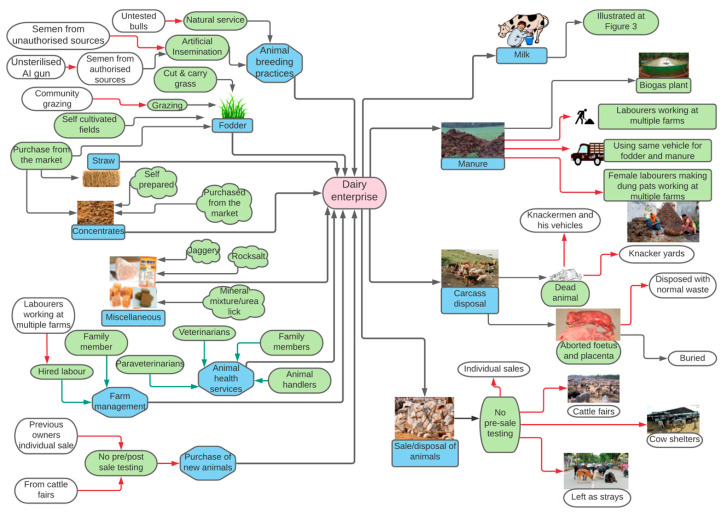
Overview of dairy input and output value chains created based on seven focus group discussions (FGDs) and five key-informant interviews (KIIs) conducted with 119 stakeholders in the Punjab state of India, 2018–2019. Green and red arrows indicate low and high risk for disease transmission, respectively.

The study locations in the state of Punjab, India are shown in Figure 1. The components of the input and output value chain towards milk production in a dairy farm are depicted in Figure 2.

### 3.1. Participants

A total of seven FGDs were conducted: Three with veterinary practitioners (41 veterinarians and 15 paraveterinarians), three with farmers (46) and one FGD with veterinary academics (12). The demographics of the participants are presented in Table 1. The majority of the participants were male (93%), middle-aged (68%) or worked in rural areas (75%). All the academics were doctorates, some practicing veterinarians held a Master’s qualification (29%) while all paraveterinarians were diploma holders. Most academics (67%) and practicing veterinarians (44%) had 10–20 years of experience, while most paraveterinarians (60%) had experience of less than 10 years. While most of the veterinarians and paraveterinarians lived (61% and 80%) and worked (71% and 80%) in rural areas, respectively, all veterinary academics lived in an urban area.

The farmers (39%) comprised the largest group of the participants of the FGDs, with more than half (63%) belonging to 20–30 years age group. A majority of farmers worked in rural areas, although 15% lived in urban areas. The number of animals reared by the participating farmers ranged from 1 to 312 dairy animals. Less than half (46%) of the participant farmers named livestock farming as their main occupation, while crop cultivation (20%) and other occupations (17%) took precedence over livestock farming for others. One third of the farmers had experience of 21–30 years in livestock farming, while less than half (46%) had been in livestock farming for less than 10 years. Most of the farmers were smallholders and owned a median of five bovines.

### 3.2. Input Value Chains

#### 3.2.1. Feed

The feed, which comprised of fodder (green and dry fodder), concentrate feed and a mineral mixture, was reportedly the most expensive input of the value chain.

1. Green fodder: The farmer FGDs revealed that on average, 82% of farmers self-cultivated their green fodder, while others either purchased (12%), or harvested uncultivated grass or allowed their animals into community grazing fields (6%). The veterinary practitioners’ assessment regarding the supply of green fodder varied slightly from the farmers’ FGDs. They believed that a higher proportion of farmers (92%) self-cultivated fodder and only 6% purchased green fodder. The percentage of farmers allowing community grazing according to the veterinary practitioners was reported to be similarly low (3%; Table 2).
2. Dry fodder: It was reported that the dry fodder usually consisted of wheat straw, which was obtained from the remains of the wheat crop harvest. The farmer FGDs reported that on an average 60% of the farmers used the wheat straw from their own fields, while 40% purchased it from the market, a figure that was found to be similar to the results of the questionnaire data (63% self-cultivated and 37% purchased). The veterinary practitioners, however, felt that a larger proportion of the farmers (75%) used the self-cultivated wheat straw (Table 2).
3. Concentrates: Majority of farmers (63%) reported to purchase the readymade cattle product for the concentrate feed during the FGDs, but the proportion was slightly lower (55%) in the individual responses to the questionnaire. The average assessment of the veterinary practitioners was similar to the farmer FGDs. A large majority (75%) of farmers reported not using mineral supplementation in their animal feed, which was contrary to the findings of the questionnaire (47%) or the assessment of the veterinary practitioners (20%; Table 2).

#### 3.2.2. Artificial Insemination and Veterinary Services

The veterinarians and paraveterinarians were the most sought-after by the farmers for most veterinary advice and interventions (Table 3). They were most approached when seeking general advice, which includes verbal consultation about livestock feeding, breeding and management (veterinarians 60%; paraveterinarians 20%), but others (animal attendants and inseminators) were equally sought-after for minor veterinary interventions (general cases) or procedures such as “retention of placenta”, “assisting in calving” or “handling abortions”. No veterinary certification for the animals was reportedly required for the purpose of purchasing or introducing new animals.

The veterinary and the animal health services for the dairy animals included: artificial insemination, pregnancy testing, treatment, vaccination, dehorning and castration. The respondents attributed reliance on the paraveterinarians and animal attendants for veterinary help due to their availability and proximity. In addition, one of the practicing veterinarians admitted, “24 h availability of the unqualified individuals is the major factor for the farmers to call them (…to attend to sick animals…)”. Another reason that was cited for seeking unprofessional help by the farmers was the lower fee charged by them compared to the veterinarians, as observed by a veterinary academic: “These unqualified persons charge lower fees than the veterinarians and sometimes even collect the fee after every cash crop”.

The government veterinary services were found to be favored for vaccinations and artificial inseminations, but unqualified help was often used for other services (Table 3). Out of the government veterinary workforce, the majority of the services were reportedly provided by paraveterinarians, followed by veterinarians and animal attendants (Table 3).

During the FGDs, the majority of farmers (76%) reported getting their animals vaccinated against foot-and-mouth disease (FMD) and hemorrhagic septicemia (HS) but only a small proportion (3%) were aware of or used the brucellosis vaccine. A farmer admitted “I came to know about the brucellosis vaccine only during this training”. Another farmer observed “Brucellosis vaccination is being practiced in only the large and organized farms”. As many as 41% of the farmers reported at least one incidence of abortion at their farm during the previous two years. Most farmers (82%) claimed to have assisted during calving at least once and 34% of them had assisted in as many as six or more calvings during the past year. The majority (85%) of the farmers reportedly used artificial insemination (AI) in cattle, but the proportion favoring AI in buffaloes was comparatively smaller (68%).

### 3.3. Output Value Chains

An overview of output value chains created in the study is presented in Figure 2.

#### 3.3.1. Milk

**Figure 3:**
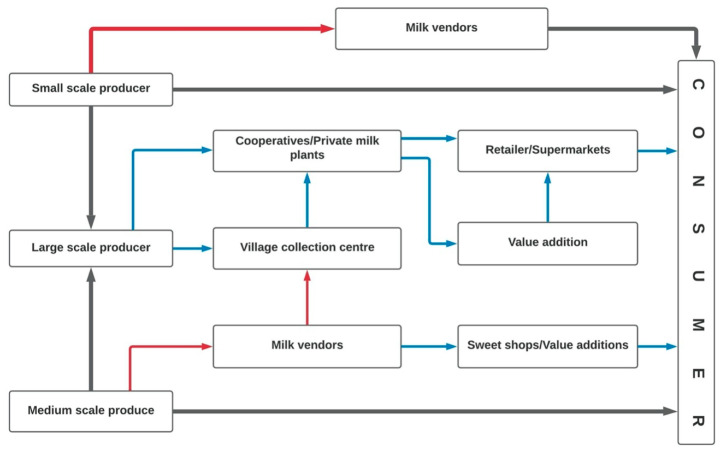
Flow diagram of the output chain of milk through various marketing channels as created during the study of dairy value chain in Punjab, India during 2018–2019. Blue and red color arrows indicate low and high risk of disease transmission, respectively.

Different chains of milk marketing are depicted in Figure 3.

A majority of the farmers (80%) reported that they supplied milk to the collection centers in the village (private or cooperative), although some (20%) sold directly to the consumers or to the milk vendors (Table 4). It was reported that the vendors sold milk to consumers going door-to-door in nearby towns or cities or supplied in bulk to the milk product manufacturers.

Fewer than 5% of the farmers reported the consumption of fresh raw milk. The milk was mostly processed to make khoa (dried or thickened whole milk), cottage cheese (paneer) and yoghurt for personal use or for selling. It was mentioned during the FGDs that commercial dairy farmers delivered their milk directly to cooperative or private processors, but some of them had started manufacturing dairy products themselves, recognizing the extra value that could be gained in addition to the raw milk.

#### 3.3.2. Animal Sales and Culling

**Figure 4:**
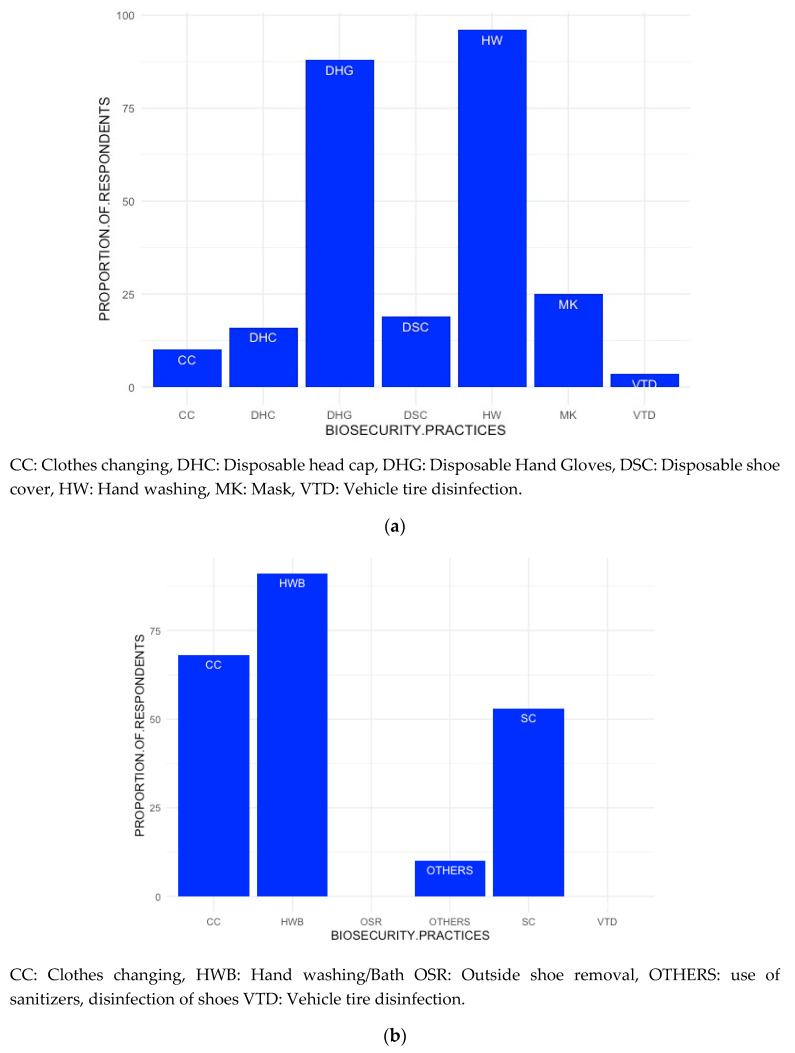
(**a**) The proportional distribution of the different biosecurity practices adopted by the veterinary professionals (veterinarians and paraveterinarians) while moving from farm to farm based on the responses received during dairy value chain study conducted in Punjab, India during 2018–2019; (**b**)the proportional distribution of the different biosecurity practices adopted by the veterinary professionals (veterinarians and paraveterinarians) on returning home from farm visits based on the responses received during dairy value chain study conducted in Punjab, India during 2018–2019.

Although 15% of the farmers confirmed keeping farm records, only one (2.5%) of them would ask for or provide health certification during sale and purchase of dairy animals, and only a few farmers (6%) said that they would ask for testing of the animals for brucellosis or any other diseases before sale or purchase (Figure 4). During the FGDs most farmers mentioned that the unproductive buffaloes were sold through brokers, who then supplied them to slaughterhouses, either directly or through mediators. Farmers mentioned that the state ban on cow slaughter has led to old, unproductive cows and male calves being abandoned to stray in the streets. It was mentioned in one of the FGDs that the newborn male cattle calves were left to die, because they are useless for draught purposes in the wake of mechanization of agriculture.

#### 3.3.3. Carcass Disposal

It was revealed during farmer FGDs that animal carcasses are disposed of at sites (knacker yards) designated for this purpose for a cluster of villages generally located outside village premises. The knackers carry the dead animals to the disposal site and leave the deskinned carcass to decompose or to be scavenged, mostly by free-roaming dogs. In the case of insured animals, post-mortem examination is also conducted by the veterinarians at such sites and the remains are left for the scavengers. Most farmers reported that they bury aborted fetuses or placentas, but some farmers discarded them with normal waste if the fetus was small (Table 4). To quote a farmer from one of the FGDs, “If aborted fetus is small, then we usually throw it away, otherwise bury it”.

#### 3.3.4. Biosecurity Practices

The biosecurity practices adopted by the veterinary professionals after performing a veterinary procedure are presented in Table 5. To perform procedures such as pregnancy diagnosis, attending to abortion cases, resolving retention of placenta, or inseminating a cow, it was found that most veterinary professionals would wear disposable gloves (>90%), but not an apron, masks or goggles. Relatively, a higher number (26%) would assist in calving without wearing gloves. Generally, the frequency of the use of aprons, goggles and mask was found to be lower than gloves and washing of hands after attending to a clinical case.

The biosecurity procedures adopted by the veterinary professionals while moving from one farm to another are illustrated in Figure 4a. Practices such as changing of clothes (10%), use of disposable shoe covers (19%), head caps (16%) or masks (25%) or disinfection of vehicle tires (3.5%) while travelling between different farms were adhered to by few professionals. A larger proportion of veterinary professionals, however, performed some kind of protection when returning home from a farm visit (Figure 4b). More than half (60%) would either remove their work shoes or clean their shoes (53%) prior to entering their houses, while a larger majority would change working clothes (68%) or perform a thorough hand wash or take a bath (91%) after returning home from work (Figure 4b). Some veterinary professionals reported that they use sanitizers or disinfectants (three respondents each) or disinfect their shoes (one respondent; Figure 4b).

**Figure 5:**
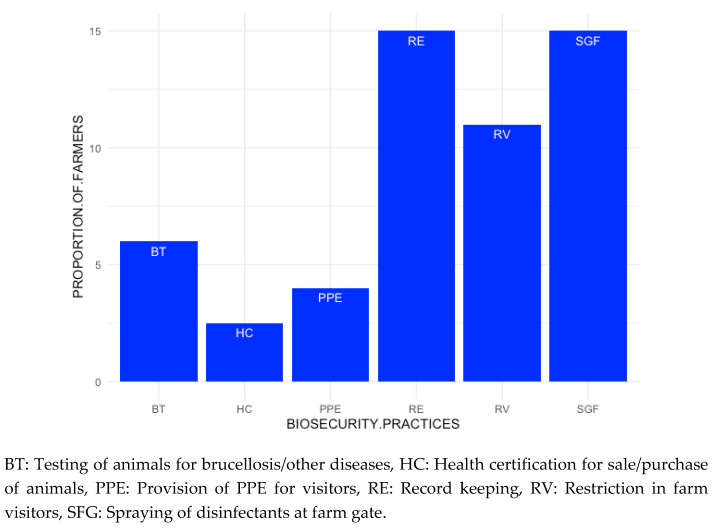
The proportional distribution of the different farm biosecurity practices adopted by the farmers based on the responses received during dairy value chain study conducted in Punjab, India during 2018–2019.

The personal biosecurity practices observed by the farmer participants such as the use of personal protective equipment (PPE) was found to be negligible, as calving or dystocia cases were reportedly handled with bare hands without sleeves or gloves (Figure 5). Only 4% of the farmer respondents reported that they provide any kind of PPE to the visitors to their farm. While during the FGDs the farmers did not report any restriction on the human traffic visiting the farm (guests, relatives, neighbors, friends, relatives, brokers, salesmen, service providers or farriers), 11% of them answered that they do so in the questionnaire. Few farmers (15%) reported to have disinfectants sprayed at the farm gates. The personnel working at multiple farms reportedly moved freely between farms without observing any biosecurity measures. Further, it was reported that the bulls used for natural services at the village level were rarely tested for brucellosis or other diseases and the available laboratories reportedly remain underutilized. One participant (farmer) revealed that “Artificial insemination is the first choice but in case the animal does not conceive, then we opt for natural service”.

#### 3.3.5. Manure Disposal and Use

The FGDs revealed that biosecurity measures were seldom performed during animal handling and management. For example, it was found that the farmers used the same carts/vehicles to ferry manure out of the farm and to bring in fodder to feed the animals. As one farmer said, “I have only one cart, every day I use to carry the dung from the dairy farm to crop fields to use it as manure and bring back the fodder on same cart from crop fields to dairy farm”.

The FGDs revealed that the cow dung was also used to coat the cleaned earthen floors of the households, a task that was exclusively carried out by the female members of the household.

Farm chores such as dung-cake preparation (100%) and manure management (70%) were mostly performed by female farmers, while other chores such as purchase of animal feed, feeding of animals and manuring of the farms were predominantly carried out by the male farmers. Women also contributed to milking the animals (60%) and cleaning of milk utensils (50%; Table 6). A majority of farmers (67%) favored using the dung as fuel for which it was converted into dried cowpats, while the rest of the farmers (37%) utilized the collected dung heap as manure for their fields (Table 6).

## 4. Discussion

This study was conducted to describe the existing dairy value chains in India and identify the vulnerable links that facilitate the spread of infectious diseases. While some positive farmer practices were identified, the study also identified links and practices that need change to improve farm productivity and to protect dairy animals from diseases. The study also highlighted the benefits of using the bottom-up approach for understanding the dairy industry. This approach may find utility in other animal industries in India and in other developing countries, to identify vulnerabilities in value chains and to develop context-based solutions.

### 4.1. Input Value Chains

#### 4.1.1. Feed

An optimum mix of traditional fodder, enriched concentrate feed and mineral mixture that meets the physiological needs of a dairy animal (in-milk, dry, pregnant or heifer) is paramount to ensuring adequate intake of protein, energy, minerals and vitamins [7]. Not surprisingly, it constitutes the most expensive component of the dairy value chain.

1. Green fodder: Punjab is one of the better placed Indian states in terms of green fodder production due to its favorable agroclimatic conditions [19]. However, 18% of farmers relied on purchased green fodder or leaving their animals to community grazing. A dairy animal on average requires at least 40–50 kg of green fodder per day (8–10% of body weight) [20]. The daily availability of green fodder in the state varies from 25.6 to 28.1 kg per adult cow [21] indicating deficiency of green fodder, resulting in nutritional deficiencies and inadequate milk production [22]. The shortage of green fodder has been amplified over years due to the increased pressure on cultivable land to grow food grains, pulses and oilseeds to cater for growing human population in India. To bridge the gap between the demand and availability of fodder, the government of India added a feed and fodder component to the national livestock mission (NLM) [3]. The mission aims to enhance fodder production in the country by introducing fodder crops in the crop rotation, developing regional fodder calendars, organizing the demonstration of fodder conservation methods and establishing training programs for the farmers, among other related initiatives. Self-cultivated fodder emerged as a crucial link in the value chain, and implementation of such objectives would enhance the availability of green fodder to dairy farmers.It is pertinent to understand that the NLM drive follows earlier initiatives of the Feed and Fodder Development (FFDP-2005/06) and Accelerator Fodder Development Program (AFDP-2011/12), which largely remained ineffective [4,23]. Hence, the success of the NLM objectives will much depend on bringing radical changes to the farm practices through awareness campaigns and training.
2. Dry fodder: Wheat is a major cereal crop in Punjab and use of the crop-residue for livestock feed is not unexpected [24]. However, a major proportion of the respondents (37%) reportedly purchase wheat straw from the market to feed their animals. This implies that either the crop residue obtained is grossly inadequate or is unavailable to small scale livestock farmers with small or no land holdings. Interestingly, in contrast the state faces a problem of underutilization of crop residues, and cultivators resort to crop burning, causing enormous environmental pollution [25]. Unfortunately, a study conducted to explore sustainable solutions to limit the burning of crop residue did not rank its usage for animal feed as a priority [26] against other economically beneficial options. The crop residue management scheme announced by the Government of India is likely to address this issue by encouraging the use of crop residues for environmentally friendly options such as a source of animal feed [26,27]. A major gap noted in the farmer FGDs is that silage making practices were not mentioned, although silage has been recommended by nutrition experts as a substitute when green fodder is not available [28]. The use of silage minimizes the total loss of nutrients from harvest through storage and offer increased feed handling efficiency [29], in addition to increasing the efficiency of land use [30]. However, despite the efforts of extension agencies [31], the uptake of this practice in this study was found to be negligible. Presumably, this may be due to poor awareness among farmers, low land availability or priority of other cash crops. Another factor that might restrict this activity is the enormous labor input involved in silage making, which makes it economically unviable for small-scale farmers. However, the barriers to the adoption of this practice need further investigation. A notable finding of the FGDs is that fewer farmers (<5%) opted for community grazing practices. There has been a reduction in the availability of open spaces for grazing. This may be because available space is increasingly being utilized for the cultivation of commercial crops, which restricts cattle grazing [32]. While this reduces the risk of animal-to-animal transmission of infectious diseases, it severely compromises welfare by restricting animal movement. The prospect of increasing farm sizes to facilitate grazing seems improbable in the future, but the option of erecting exercise rings within the available area could be explored. The practice of community watering at communal ponds was also found to have disappeared over years [33], however, the stakeholders of this study were unanimous that this practice is no longer critical. Most of the farmers use bore water or have installed submersible pumps for their watering needs. This is a positive development for animal and public health as animals are less likely to contract infectious diseases than they would have while congregating at the pond. Additionally, they are less likely to be infected with parasites such as liver fluke, which requires intermediate hosts such as snails living in or near ponds.
3. Concentrates: In contrast to the practice in the rest of the country, where concentrate feed constitutes a mere 6% of the feed composition of dairy animals, the farmers in Punjab were reportedly more inclined to use compound or concentrated animal feed, as most of them purchased the concentrate [4]. The practice of using concentrate feed has in fact risen to 60% from less than half of the farmers in the state as reported by Janssen and Swinnen [19]. This could be due to better reach of the dairy cooperatives and multinational companies such as Nestle in Punjab compared with other states in India [19]. Nonetheless, a substantial majority of the respondents in this study (FGD average—37%, individual response—45%) still used self-prepared concentrate feed, which is possibly a reason for the milk production being below potential (Table 2). We understand that the practice of self-preparing feed concentrate consisting of on-farm produce mixed with locally available ingredients is more characteristic of small-scale farmers. These farmers refrain from purchasing commercial cattle feed due to economic constraints [4,34]. The farmers who purchased concentrate feed expressed dissatisfaction with the varying quality of the concentrate available in the market [35]. While it would be desirable for large scale dairy farmers to make their own feed mixes, educating farmers on the constituents of a good concentrate feed may not be practically viable. Hence, we recommend that the regulatory body constituted by the government must strictly ensure adherence to minimum standards of feed quality in the open market.
4. Mineral mixtures: Surprisingly, according to the individual farmer response to the questionnaire, the usage of mineral mixture in the area of study has doubled compared to a study conducted by Bakshi and Wadhwa [34]. The use of mineral mixture is important in order to maintain reproductive potential and to prevent anestrous and infertility in cows. The increase in its usage could be credited to the success of extension messages conveyed by the government agencies in convincing farmers about its benefits. Nonetheless, there remains a huge potential for wider use of mineral mixtures in dairy animal feed, which could improve animal reproductive health and milk production.

#### 4.1.2. Veterinary and Animal Health Management Services

Provision of an efficient veterinary coverage to dairy livestock including vaccination against infectious diseases is a key link in the value chain to improve milk production. Three types of service providers were included in the FGDs: (a) qualified registered veterinarians, (b) paraveterinarians and (c) animal attendants and others such as inseminators. Although the inseminators and animal handlers are trained only to perform artificial insemination and pregnancy diagnosis, they are often approached by farmers to perform veterinary procedures beyond the scope of their qualifications. The government animal health and management services should be the main actors for provision of this crucial service, but this study revealed that a very high proportion of farmers rely on either paraveterinarians or animal handlers/inseminators for health advice and veterinary procedures. These included artificial insemination, pregnancy diagnosis, abortion handling, calving assistance and treatment of retention of placenta. These service providers, including the veterinarians, have limited training on farm biosecurity and may possibly be responsible for the spread of infections, although there are no studies to support this contention. Chandel and Singh [36] advocated a government policy intervention in this regard to enable farmers to better utilize the services provided by the state actors as they observed that despite the state veterinary services being free of cost, the expenditure involved for movement of animals to government hospitals and follow-up visits compels the farmers to seek unqualified help. The use of veterinary ambulatory services could also be a viable option to provide the door-step help. Non-governmental registered veterinary practitioners also provide services, but usually at a higher cost, and are thus not accessible by smallholders [36]. This implies that the utilization of unqualified service providers is basically driven by economic constraints, and highlights failure of the government services to deliver doorstep services to the smallholder farmers.

This study also highlights that farmers prefer paraveterinarians to veterinarians because the former are usually more accessible, as they live in closer proximity (80% of the paraveterinarians resided in rural areas compared to 60% of the veterinarians), charge lower fees and can provide services at the farmers’ doorstep. The paraveterinarians are not trained or legally permitted to perform many veterinary interventions. It is likely that paraveterinarians would also be responsible for spread of infections between animals and farms. This is in addition to poor outcomes for the animals and the farmers in the long run. Proch, et al. [37] demonstrated that compared to the veterinarians, the paraveterinarians and animal attendants are more likely to be *Brucella* infected as the latter attend interventions more frequently, lack adequate skills and are also not fully aware of the risks involved. The prevalence of brucellosis infection in veterinary practitioners including veterinarians as reported elsewhere reinforces the finding that the necessary biosecurity measures are inadequate [38,39,40]. While the role of paraveterinarians in delivering veterinary services is crucial, especially in the rural areas as in many other developing countries, there needs to be a clear demarcation of their role from that of veterinarians. Alternatively, their veterinary training must be scaled up to include biosecurity, disease management and infection control along with the importance of the use of personal protection. Provision of PPE for veterinary use, training of personnel for its use and reinforcing its regular use by practitioners also demands serious attention on the part of policy makers, extension services and state veterinary department alike.

Holistically, biosecurity practices at the dairy farms in the study areas were found to be negligible. Animals are not tested before introduction to the farm (or when sold or culled), no quarantine measures are followed, and there are no regulations in place to restrict movement of animals or visitors including veterinarians and paraveterinarians. No farmers reported using any PPE, even when assisting in parturition procedures, or handling fetuses or placenta. Although such omissions are not totally unexpected in rural India, this underlines the gap in knowledge, awareness and practice regarding the importance of observing farm biosecurity amongst farmers, paraveterinarians, animal handlers and veterinarians.

### 4.2. Output Value Chains

#### 4.2.1. Milk

Most of the fresh milk is sold to consumers directly or through milk vendors without pasteurization. Nonetheless, the practice of consuming raw milk in urban areas is rare in India with most consumers boiling the milk before consumption [41], and hence does not pose any public health concerns. However, one key informant revealed that they prepare milk products such as cream from raw milk. This cream is further used to prepare other milk products for which the ingredients are either boiled or heated, likely killing the disease-causing pathogens. However, the raw cream could possibly be consumed, which has a potential risk of transmitting zoonotic and food-borne pathogens. We recommend a thorough evaluation and risk analysis of the milk processing value chain to identify milk products that may pose a risk of contamination by food-borne pathogens. Further, educational and training programs should be developed to encourage small-scale milk-product manufacturers to prepare cream from only pasteurized milk.

The FGDs revealed that the government agencies encourage farmers to add value to their raw milk to enhance income. However, the lack of a structured programme that could educate farmers in healthy practices and regulate procedures towards safe value addition has prevented most farmers from benefiting from such government initiatives [19].

#### 4.2.2. Animal Purchase/Sale and Culling

The lack of regulations for purchase of dairy animals with regard to their health, and reproductive and productive fitness is a long-standing shortcoming of dairy husbandry practices in India that was revealed in this study. As sale or purchase of dairy animals is profit-driven, it is only expected that sellers do not disclose any information that would hamper or reduce sale value. Surprisingly, studies reveal that the requirement of health certification or proof of testing against infectious diseases does not appear among the list of qualitative and quantitative factors considered to affect the market price of murrah buffaloes, milch cows and bullocks [42,43,44]. The digitalization of animal husbandry practices including sale or purchase of animals could address this issue, as it could facilitate tracing of the animal movements as they change hands [45,46]. In fact, an effective Information and Communications Technology (ICT) backed system could enable real-time identification of supply chains in dairying and other animal industries [47]. Some examples of such systems are the ones that are followed in Australia [48], Denmark [49] and New Zealand [50] among others.

The study also brought to light the plight of the existing cow-shelters (gaushalas) that have witnessed a heavy influx of old or expended dairy cows following the state ban on cow-slaughter [51]. Hindus who form the majority religious group in India, consider cows to be sacred and therefore cows are not allowed to be slaughtered in most states. Cow shelters have been set up by the government and some social and religious bodies to house such unproductive and senile cows, but their capacity is limited. Moreover, gaushalas charge the cattle owners a fee, which is not affordable by many smallholding farmers. As the gaushalas receive more cattle that they can accommodate it is predictable that most farmers let their culled animals stray on the streets, which facilitates transmission of zoonotic diseases such as brucellosis and rabies [52,53]. Although unproductive buffaloes are sold to slaughterhouses via brokers, cows cannot be sold and thus become stray cattle resulting in traffic accidents [54]. While the cow-slaughter ban is unlikely to be lifted by law in the near future, the following actions are recommended: establish more shelters for culled cattle, improve the management of the existing shelters and enhance funding for animal welfare. There should also be a wider application of assisted reproduction technologies (ART) such as sex preselection techniques in producing and distributing sexed semen [54,55,56].

#### 4.2.3. Carcass Disposal

An important aspect of the output supply chain that was highlighted during this study is the disregard paid to the disposal of carcasses. Unfortunately this practice has not improved from the age-old system when carcasses were left in the open for scavenging birds such as vultures [57,58]. With the rapid fall in vulture populations, the remains of these dead dairy animals are eaten by dogs [59], which are potentially infected with brucellosis [60] and other zoonotic diseases such as rabies [53]. These dogs can in turn be responsible for the spread of these diseases to other healthy livestock, dogs or even humans [61]. We recommend further research to explore the potential spread of infection through diseased carcasses.

The dumping of carcasses at designated areas in the open is an environmental nuisance, but ignorance of biosecurity measures by individuals deskinning the dead animals in the knacker yards is also a grave public health hazard [62]. Further, the knackers do not use PPE while transporting, handling and deskinning the dead animals, which possibly exposes them to high risk of contracting zoonotic diseases similar to that of abattoir workers. We recommend studies to investigate the prevalence of zoonotic diseases in knackery workers and their families to better understand the magnitude and frequency of such transmissions. Unfortunately, the knackers are usually uneducated and belong to marginalized sections of the society, with limited or no influence over public policy or research funding [63]. It is imperative that government institute awareness programs to educate them on personal protection and disease control measures, and to train them to use PPE [64]. Additionally, the casual attitudes and practices of dairy farmers toward disposal of aborted fetuses and placenta could be exposing free-roaming scavenging dogs to potential infections including brucellosis [37]. Surprisingly, there are few awareness programs for the farming community to inform them of such potential hazards. A government policy to regulate the disposal of carcasses of not only dairy animals but also other livestock will go a long way to providing tangible solutions. Ahuja [62] elaborated cost-effective methods of carcass disposal for India. It is recommended that a government facility comprising a post-mortem room and incinerator where the carcasses could be destroyed should be established to solve this vulnerable component of the dairy value chain.

#### 4.2.4. Manure Disposal and Use

A number of studies demonstrate that handling animal manure is a risk factor for brucellosis in humans [39,65]. The FGDs revealed an absence of farm level hygienic practices, which possibly not only facilitates the high prevalence of brucellosis in animals and humans, but also other bacterial pathogens such as *Campylobacter jejuni* and pathogenic *Escherichia coli* [66]. Women and girls are presumably exposed to such risk more often than male farmers, as the making of cowpats is a task done exclusively by them. This is, however, in contrast to a study of tribal women from the state of Gujarat, India, where only 67% of women participated in animal manure disposal, while less than half (44%) participated in dung cake preparation [67]. Although clinical profiling studies in other parts of India show a significant male prevalence of brucellosis [68,69], we understand that there may be an under-reporting by female patients from rural areas to clinics, which explains such observations. Community based cross-sectional studies can provide more information regarding any association between women handing animal manure and the incidence of related infections.

Further, using the same cart for disposal of animal manure followed by carrying fodder intended for animal consumption may lead to contamination of fodder and bedding materials. This practice could be a possible cause of a number of diseases including zoonoses. Farmers used negligible PPE during their routine farm operations. We recommend that in addition to spreading awareness regarding the use of PPE, it is essential to make such provisions affordable and available. For example, a study by Biswas, et al. [70] demonstrated a high incidence of skin ailments in dairy farmers, including women, due to the handling of animal manure. However, the workers seldom use gloves because they are either not available or are unaffordable.

The disposal of dung and effluent is an important component of the output value chain. The government extension agencies have been encouraging farmers to install biogas plants that use dung as input and produce biogas and nutrient-rich sludge. The biogas can be used as fuel in the kitchen and the sludge can be used as fertilizer for crops. A green source of energy, it reduces reliance on fossil fuels and is beneficial for the farmer and for the environment. However, our results suggest that the uptake of this recommendation is very poor. A study by the Indian Institute of Technology, Bombay, identified economic and cultural issues as hindrances to the spread of biogas technology in the Indian rural diaspora. This is in spite of a monetary subsidy being available for the installation of the plant [71,72]. The barriers for poor uptake of biogas technology in Punjab need to be investigated further.

### 4.3. System Vulnerabilities for Infectious Disease Transmission and Spread

A fundamental weakness of the Indian veterinary system lies in the uncontrolled movement of animals, whether for seeking veterinary care, sale or purchase, as the risk of spreading diseases through such widespread movement is undoubtedly high. The delivery of veterinary services at the doorstep is either available at a premium beyond the reach of smallholding farmers or is provided by unqualified persons, who instead inadvertently help to propagate infections. Although there are diagnostic facilities developed by the government at the district and subdistrict level, a majority of the laboratories remain underutilized. This is apparently due to the shortage of trained professionals and a gap in epidemiological knowledge. An epidemiology capacity building program would help to enhance the expertise of veterinary professionals to effectively utilize available laboratory facilities for the advancement of animal health. We also recommend a structural change in the delivery of veterinary services; providing mobile veterinary help could be considered in this regard.

The current study describes the dairy value chains in the Punjab state of India and identifies their vulnerable links. The dairy value chain in the rest of the country is not much different to that found in this study. We believe that the identified risk nodes in dairy production in Punjab should be considered while recommending biosecurity practices. In addition, these identified risk nodes will inform disease prevention and control in other parts of the country. However, we identified some limitations in this study. Firstly, the results of the questionnaire responses sometimes did not align with the outcome of the FGDs. We believe this relates to the reluctance of the farmers to reveal their concerns in open discussion. Secondly, the opinions of the farmers may be biased as they were selected while participating in the trainings conducted by GADVASU. They may be more informed than other farmers from remote locations who could not afford to travel to the university agriculture training programs. Another omission of this study is exclusion of the biosecurity measures during harvesting of milk. We recommend including this crucial aspect for future studies. Finally, although anonymity was ensured, the veterinary practitioners may have introduced some bias, possibly being guarded in their responses, as they were all government employees.

In spite of these limitations, the current study highlights nutritional, managemental, biosecurity and regulatory issues in the input and output dairy value chain in Punjab, which require attention to enhance the dairy production efficiency of the state. The study identifies several risk nodes such as inadequacy in the provision of veterinary services, improper carcass, placenta and excreta disposal, negligible use of PPE by farmers and veterinary professionals alike, and poor adherence to biosecurity practices. Improved delivery of veterinary services, availability and affordability of quality nutrition and regulations enforcing strict biosecurity farm practices including those for disposal of farm-waste and dead animals are recommended to increase dairy production and to reduce zoonotic disease transmission risks.

## 5. Conclusions and Future Perspective

This study was conducted to describe the structure of the dairy industry in the Punjab state of India and to identify practices increasing the risk of transmission of infectious diseases. The study identified several positive practices that should be continued or encouraged and some other practices that should be discontinued or discouraged. The provision of veterinary services by traditional practitioners, improper carcass/placenta/excreta disposal, limited use of personal protection, limited use of health certification during sale or purchase and limited testing of bulls were major practices increasing the risk of transmission of infectious diseases. Improved nutrition and stringent biosecurity practices are required in order to increase dairy production and to reduce zoonotic disease transmission risks.

Further epidemiological studies or risk analyses should be conducted to confirm and quantify the risks identified in this study. In particular, it is important to investigate the behavioral reasons for farmers undertaking some practices. Is this due to a lack of knowledge and awareness of the correct approach or due to other social, behavioral, cultural and financial issues? If it is just due to a lack of awareness, then educational campaigns can be conducted to improve their practices. However, if it is due to other behavioral reasons, social scientists should be involved to identify the barriers for the adoption of correct approaches. Animal health policymakers and the dairy industry should consider how the production constraints and biosecurity risks identified in the study can be mitigated to improve production, reduce the incidence of infectious diseases, increase farmer income and improve animal welfare.

## Figures and Tables

**Table 1:** Demographic characteristics of 119 stakeholders participating in seven focus group discussions (FGDs) and five key-informant interviews (KIIs) in the Punjab state of India in 2018–19.

Variable	Categories	Veterinary Academics (*n* * = 12)	Veterinarians (*n* * = 41)	Paraveterinarians (*n* * = 15)	Farmers (*n* * = 46)	KIIs (*n* * = 5)	Total *N* (%) *N* ^#^ = 119
Sex	Male	10	38	15	43	5	111 (93)
	Female	2	3	0	03	0	8 (7)
Age ^$^	20–30	1	3	3	29	1	37 (31)
	31–40	3	14	8	10	2	37 (31)
	41–60	8	24	4	6	2	44 (37)
	>60	0	0	0	1	0	1 (1)
Residence	Rural	0	25	12	39	5	81 (68)
	Urban	12	16	3	7	0	38 (32)
Working area	Rural	0	29	12	46	2	89 (75)
	Urban	12	3	0	0	2	17 (14)
	Both	0	9	3	0	1	13 (11)
Education	10th	0	0	0	10	2	12 (10)
	11–12th	0	0	0	25	1	26 (22)
	Diploma	0	0	15	0	1	16 (14)
	Bachelor	0	29	0	11	1	41 (34)
	Master	0	12	0	0	0	12 (10)
	PhD	12	0	0	0	0	12 (10)
Main Occupation	Cropping	0	0	0	9	0	9 (8)
	Livestock farming	0	0	0	21	4	25 (21)
	Mixed	0	0	0	7	0	7 (5)
	Government	12	41	15	1	1	70 (59)
	Other	0	0	0	8	0	8 (7)
Experience ^$^	1–10	4	10	9	21	1	45 (38)
	11–20	8	18	3	1	2	32 (27)
	21–30	0	9	1	16	2	28 (23)
	>30	0	4	2	8	0	14 (12)

*n* * = numbers; *N* ^#^ = total numbers; ^$^ in years.

**Table 2:** Input value chain component of feed and fodder displaying the proportion (%) of various input types based on the focus group discussions (FGDs) conducted in Punjab, India in 2018–2019.

Input Parameter	Variable	Farmers		Veterinary Personnel
		FGD Consensus Average * (*n* = 3)	Questionnaire Data Average (*n* = 46)	FGD Consensus Average (*n* = 3)
Fodder	Self-cultivated	82	87	92
	Purchased	12	13	6
	Cut-and-carry grass (uncultivated)	6	0	-
	Community grazing ^#^	4	3	3
Wheat straw	Self-cultivated	60	63	75
	Purchased	40	37	25
Concentrate feed	Self-prepared	37	45	37
	Ready-mix purchased	63	55	60
Mineral mixture	Supplementation	25	53	80
	No supplementation	75	47	20

* The “FGD consensus” indicates the average of the three consensus values reached during FGDs, whereas “questionnaire data” represents the summary from the self-completed questionnaires by FGD participants. Farmers were asked about their own use of feed and fodder, whereas veterinary personnel were asked about their assessments about the use of these products in their area of practice; ^#^ community grazing is the practice of allowing livestock to graze on a common grazing land/pasture.

**Table 3:** The mean, median, minimum and maximum interquartile proportions of likely involvement of various veterinary service providers for advice and interventions for animal health services based on the responses to the questionnaire survey obtained from veterinary practitioners employed with the Department of Animal Husbandry, Government of Punjab during the study (2018–2019).

Parameters	Veterinary Professionals	Min	Q1	Median	Q3	Max	Mean ± SD
General advice	Veterinarian	10	30	60	70	100	53 ± 26
	Paraveterinarian	0	20	20	30	60	26 ± 14
	Animal attendant *	0	0	10	10	50	8 ± 9
	Other ^@^	0	0	5	20	70	13 ± 17
General case	Veterinarian	0	20	40	50	100	37 ± 23
	Paraveterinarian	0	30	30	40	70	34 ± 14
	Animal attendant *	0	5	10	20	60	14 ±12
	Other ^@^	0	0	10	25	70	16 ± 19
Artificial insemination	Veterinarian	0	10	20	50	100	30 ± 24
	Paraveterinarian	0	25	35	40	70	35 ± 16
	Animal attendant *	0	2	10	20	40	13 ± 11
	Other ^@^	0	0	20	35	70	22 ± 21
Pregnancy diagnosis	Veterinarian	0	10	30	50	100	35 ± 27
	Paraveterinarian	0	20	30	40	70	32 ± 15
	Animal attendant	0	0	10	30	70	16 ± 18
	Other ^@^	0	0	10	25	70	17 ± 20
Abortion handling	Veterinarian	0	10	37.5	50	90	37 ± 25
	Paraveterinarian	0	20	30	40	70	32 ± 16
	Animal attendant *	0	5	10	20	60	12 ± 12
	Other ^@^	0	0	15	30	70	19 ± 19
Calving assistance	Veterinarian	0	10	40	50	90	38 ± 24
	Paraveterinarian	0	20	30	40	100	32 ± 18
	Animal attendant *	0	0	10	10	50	10 ± 10
	Other ^@^	0	0	15	30	70	19 ± 20
Retention of placenta	Veterinarian	0	10	30	50	80	30 ± 19
	Paraveterinarian	0	20	32.5	50	90	36 ± 17
	Animal attendant *	0	5	10	20	70	13 ± 15
	Other ^@^	0	0	10	40	70	21 ± 21

* Animal attendant are the helpers who provide services such as restraining the animal for veterinary examination. ^@^ Other: Inseminators.

**Table 4:** The proportions (%) of output value chains components—dung, milk and carcass disposal—based on FGDs conducted in Punjab, India during 2018–2019.

Parameter	Variable	Minimum	Q1	Median *	Q3	Maximum	Mean ± SD
Dung disposal	Dung heap/Manure	10	10	20	20	80	37 ± 38
	Dung pats	20	20	80	80	90	63 ± 38
	Biogas	0				5	2 ± 3
Milk	Milk collection center	70	70	80	80	80	77 ± 6
	Milk vendor/agent	10	10	20	20	30	20 ± 10
	Consumer	0				10	3 ± 6
Carcass	Buried	70	70	100	100	100	90 ± 17
	Thrown away	0	0	0	0	30	10 ± 17

* The “FGD consensus” indicates the average of the three consensus values reached during the three farmer FGDs.

**Table 5:** Numbers (*n*) of veterinary professionals following various biosecurity measures during and after performing a veterinary procedure based on the response to the questionnaire survey during dairy value chain study conducted in Punjab, India during 2018–2019. The respondents could choose more than one option.

Veterinary Procedures	Biosecurity Measures					
	Handwashing *n* * (%)	Disposable Sleeves/Gloves *n* (%)	Apron *n* (%)	Goggles *n* (%)	Mask *n* (%)	Nothing *n* (%)
General cases	53 (93)	32 (56)	20 (35)	10 (17)	7 (12)	1 (1.5)
Artificial Insemination	51 (89)	54 (95)	24 (42)	9 (16)	4 (7)	0
Pregnancy diagnosis	53 (93)	55 (96)	20 (35)	7 (12)	2 (3)	0
Abortion	53 (93)	54 (95)	35 (61)	17 (30)	18 (32)	0
Calving	52 (91)	42 (74)	30 (53)	12 (21)	11 (19)	0
Retention of Placenta	53 (93)	56 (98)	35 (61)	18 (32)	18 (32)	0

*n* *—number of respondents.

**Table 6:** Farmer responses: distribution (%) of dairy farm chores conducted/handled by family/hired labor and the respective gender roles obtained through farmer questionnaire survey during the study conducted on dairy value chains in Punjab, India, 2018–2019.

Job Performed	Male Family Member Only	Female Family Member Only	Hired Labor Only	All
Buy fodder/wheat straw from the market	46	0	38	17
Buy concentrate from the market	85	0	15	0
Feed animals	42	0	45	13
Manure management	11	26	58	5
Dung-cake preparation	4	43	50 *	0
Spread manure in agricultural farms	25	0	72	0
Milk animals	39	21	29	11
Deliver surplus milk to the collection center	92	0	8	0
Clean milk utensils	34	34	24	8

* All female labor.

## References

[1] Department of Animal Husbandry Dairying and Fisheries (2019). Annual Report 2018–2019.

[2] Ohlan R. (2016). Dairy economy of India: Structural changes in consumption and production. South Asia Res..

[3] Government of India (2019). Animal Husbandry & Dairying. Basic Animal Husbandry Statistics 2019.

[4] Landes M., Cessna J., Kuberka L., Jones K. (2017). India’s Dairy Sector: Structure, Performance, and Prospects.

[5] Birthal P.S., Chand R., Joshi P., Saxena R., Rajkhowa P., Khan M.T., Khan M.A., Chaudhary K.R. (2017). Formal versus informal: Efficiency, inclusiveness and financing of dairy value chains in Indian Punjab. J. Rural Stud..

[6] M4P (2008). Making Value Chains Work Better for the Poor: A Tootlbook for Practitioners of Value Chain Analysis, Version 3.

[7] Taylor N., Hinrichs J. (2012). Designing and Implementing Livestock Value Chain Studies. A Practical Aid for Highly Pathogenic and Emerging Disease (HPED) Control.

[8] Kuma B. (2012). Market Access and Value Chain Analysis of Dairy Industry in Ethiopia. Ph.D. Thesis.

[9] Carron M., Alarcon P., Karani M., Muinde P., Akoko J., Onono J., Fèvre E.M., Häsler B., Rushton J. (2017). The broiler meat system in Nairobi, Kenya: Using a value chain framework to understand animal and product flows, governance and sanitary risks. Prev. Vet. Med..

[10] Lowe M., Gereffi G., Ayee G., Denniston R., Fernandez-Stark K., Kim J., Sang N. (2009). A Value Chain Analysis of the U.S. Beef and Dairy Industries.

[11] Achchuthan S., Rajendran K. (2012). A Study on Value Chain Analysis in Dairy Sector Kilinochchi District, Sri Lanka. Glob. J. Manag. Bus. Res..

[12] Vamsidhar Reddy T.S., Baltenweck I., Kumari R., Jha A.K., Teufel N. Issues in the development of dairy value chains in rural India. Proceedings of the Presented at the International Conference of Agricultural Economists (ICAE).

[13] COI (2011). 2011 Census of India.

[14] NDDB (2014). Dairying in Punjab A Statistical Profile.

[15] NSSO (2013). Livestock Ownership in India (70th Round) Ministry of Statistics and Programme Implementation.

[16] Dhand N. General evidence for a brucellosis control program in India. Proceedings of the Society for Veterinary Epidemiology and Preventive Medicine (SVEPM).

[17] R Core Team (2019). R: A Language and Environment for Statistical Computing.

[18] Wickam H. (2016). Ggplot2: Elegant Graphics for Data Analysis.

[19] Janssen E., Swinnen J. (2019). Technology adoption and value chains in developing countries: Evidence from dairy in India. Food Policy.

[20] Singh P.M., Pande D. (1998). AB Managing Agriculture for a Better Tomorrow: The Indian Experience. Managing Agriculture for a Better Tomorrow: The Indian Experience.

[21] Singh R. (2016). Availability and Utilization Pattern of Green Fodder in Punjab. Master’s Thesis.

[22] Nagrale B.G., Datta K., Chauhan A. (2015). An analysis of constraints faced by dairy farmers in Vidarbha region of Maharashtra. Indian J. Dairy Sci..

[23] Planning Commission, Government of India (2012). Report of the Working Group on Animal Husbandry and Dairying for the 12th 5-Year Plan (2012–2017).

[24] Devi S., Gupta C., Jat S.L., Parmar M. (2017). Crop residue recycling for economic and environmental sustainability: The case of India. Open Agric..

[25] Kaur A. (2017). Crop residue in Punjab agriculture-status and constraints. J. Krishi Vigyan.

[26] Kumar P., Singh R.K. (2020). Selection of sustainable solutions for crop residue burning: An environmental issue in northwestern states of India. Environ. Dev. Sustain..

[27] Kumar P., Kumar S., Joshi L. (2015). Socioeconomic and Environmental Implications of Agricultural Residue Burning: A Case Study of Punjab.

[28] NDDB (2020). Silage Making.

[29] Mahanna W., Chase L., Dwayne R., Buxton R.E.M., Joseph H.H. (2003). Practical applications and solutions to silage problems. Silage Science and Technology.

[30] Phillips C.J.C. (1988). The use of conserved forage as a supplement for grazing dairy cows. Grass Forage Sci..

[31] Sharma M., Singh G., Keshava (2014). Impact evaluation of training programmes on dairy farming in Punjab State. Indian Res. J. Ext. Edu..

[32] Lindahl J.F., Deka R.P., Asse R., Lapar L., Grace D. (2018). Hygiene knowledge, attitudes and practices among dairy value chain actors in Assam, north-east India and the impact of a training intervention. Infect. Ecol. Epidemiol..

[33] Sharma P., Pandey S., Sharma R., Upadhyay J., Ray A., Biradar N., Radotra S. (2020). CPLR and Water Resource Utilization by Livestock Farmers in Different Ecosystems of India. https://uknowledge.uky.edu/igc/23/3-6-1/8/.

[34] Bakshi M., Wadhwa M. (2011). Nutritional status of dairy animals in different regions of Punjab State in India. Indian J. Anim. Sci..

[35] Singh J., Singh P., Verma H. (2012). Knowledge level of cattle feed manufactures of Punjab. J. Anim. Res..

[36] Chandel B., Singh R. (2015). Policy interventions for mainstreaming of small milk producers in contemporary production system-a value chain analysis of Indian dairy sector. Indian J. Dairy Sci..

[37] Proch V., Singh B., Schemann K., Gill J., Ward M., Dhand N. (2018). Risk factors for occupational Brucella infection in veterinary personnel in India. Transbound. Emerg. Dis..

[38] Mrunalini N., Reddy M., Ramasastry P., Rao M. (2004). Seroepidemiology of human brucellosis in Andhra Pradesh. Indian Vet. J..

[39] Yohannes Gemechu M., Paul Singh Gill J. (2011). Seroepidemiological survey of human brucellosis in and around Ludhiana, India. Emerg. Health Threat. J..

[40] Shome R., Kalleshamurthy T., Shankaranarayana P.B., Giribattanvar P., Chandrashekar N., Mohandoss N., Shome B.R., Kumar A., Barbuddhe S.B., Rahman H. (2017). Prevalence and risk factors of brucellosis among veterinary health care professionals. Pathog. Glob. Health.

[41] Sharma H., Jadhav V.J., Garg S.R. (2020). Aflatoxin M1 in milk in Hisar city, Haryana, India and risk assessment. Food Addit. Contam. Part B.

[42] Das G., Jain D. (2013). Research Note: Factors Affecting the Price of Bullocks in the Organised Cattle Fairs of Rajasthan. Indian J. Agric. Econ..

[43] Das G., Jain D., Dhaka J. (2014). Analysis of price spread and marketing efficiency of milch cow marketing in the state level cattle fairs of Rajasthan, India. Saarc J. Agric..

[44] Mondal P., Pandey U. (1993). Factors influencing the market price of lactating Murrah buffaloes in Haryana. Indian J. Agric. Econ..

[45] Neethirajan S. (2020). Digitalization of Animal Farming. Preprints.

[46] Hiremath Uday V. (2018). Digitalisation in Rural Entrepreneurship a Paradigm Shift. Int. J. Trend Sci. Res. Dev..

[47] Mudda K., Giddi B., Murthy P. (2017). A study on the digitization of supply chains in agriculture-an Indian experience. J. Agric. Inform..

[48] Integrity Systems National Livestock Identification System. https://www.nlis.com.au.

[49] Danish Veterinary and Food Administration Livestock Identification, Registration and Traceability. https://www.foedevarestyrelsen.dk/english/Animal/AnimalHealth/Animal%20diseases/Monitoring_control_animal_diseases/Livestock_identification_registration_and_traceability/Pages/default.aspx.

[50] Ministry of Primary Industries National Animal Identification and Tracing (NAIT) Programme. https://www.mpi.govt.nz/animals/national-animal-identification-tracing-nait-programme/.

[51] Kennedy U., Sharma A., Phillips C.J. (2018). The Sheltering of unwanted cattle, experiences in India and implications for cattle industries elsewhere. Animals.

[52] Brookes V., Gill G., Singh C., Sandhu B., Dhand N., Singh B., Gill J., Ward M. (2018). Exploring animal rabies endemicity to inform control programmes in Punjab, India. Zoonoses Public Health.

[53] Singh B.B., Kaur R., Gill G.S., Gill J.P.S., Soni R.K., Aulakh R.S. (2019). Knowledge, attitude and practices relating to zoonotic diseases among livestock farmers in Punjab, India. Acta Trop..

[54] Khan A., Riedel T., Hussain R., Patel I. (2020). Beef Ban in India: A Multi-dimensional Issue. J. Pharm. Pract. Community Med..

[55] Bijla S., Khalandar S., Sharma P., Singh A. (2019). An Analysis of Constraints Faced by Gaushalas in Haryana. Econ. Aff..

[56] Rao T., Chaurasia S., Singh A., Gamit V. (2016). Management of Stray Cattle in Urban Area. Tongue Grafting Sohiong.

[57] Cunningham A.A., Prakash V., Pain D., Ghalsasi G., Wells G., Kolte G., Nighot P., Goudar M., Kshirsagar S., Rahmani A. (2003). Indian vultures: Victims of an infectious disease epidemic?. Anim. Conserv..

[58] Taggart M.A., Cuthbert R., Das D., Sashikumar C., Pain D., Green R., Feltrer Y., Shultz S., Cunningham A., Meharg A. (2007). Diclofenac disposition in Indian cow and goat with reference to Gyps vulture population declines. Environ. Pollut..

[59] Singh B., Ghatak S., Banga H., Gill J., Singh B. (2013). Veterinary urban hygiene: A challenge for India. Rev. Sci. Tech. Off. Int. Epiz.

[60] Pal M., Gizaw F., Fekadu G., Alemayehu G., Kandi V. (2017). Public health and economic importance of bovine Brucellosis: An overview. Am. J. Epidemiol. Infect. Dis..

[61] Chugh T. (2008). Emerging and re-emerging bacterial diseases in India. J. Biosci..

[62] Ahuja S. (2011). Cost Effective Solution for Carcass Disposal in India. Int. J. Environ. Sci..

[63] Sharma R. (2017). Dalits and human rights in India. Towards Light.

[64] Pahwa S., Swain S. (2020). The fate and management of sick and dying cattle–Consequences on small-scale dairy farmers of peri-urban areas in India. Indian J. Community Med. Off. Publ. Indian Assoc. Prev. Soc. Med..

[65] Mantur B., Amarnath S., Shinde R. (2007). Review of clinical and laboratory features of human brucellosis. Indian J. Med. Microbiol..

[66] Penakalapati G., Swarthout J., Delahoy M.J., McAliley L., Wodnik B., Levy K., Freeman M.C. (2017). Exposure to animal feces and human health: A systematic review and proposed research priorities. Environ. Sci. Technol..

[67] Vahora S., Thorat G., Ramjiyani D. (2016). Involvement of Tribal Dairy Women in Health Care Management Practices of Animal Husbandry. Gujarat J. Ext. Educ..

[68] Kochar D., Gupta B., Gupta A., Kalla A., Nayak K., Purohit S. (2007). Hospital based case series of 175 cases of serologically confirmed Brucellosis in Bikaner. JAPI.

[69] Sathyanarayanan V., Razak A., Saravu K., Ananthakrishna S.B., Prabhu M.M., Vandana K. (2011). Clinical profile of brucellosis from a tertiary care center in southern India. Asian Pac. J. Trop. Med..

[70] Biswas S., Chatterjee P., Mukherjee C., Pal P., Pradhan N. (2015). Awareness of farmers Regarding hygienic handling of their cattle to prevent zoonotic diseases. Explor. Anim. Med. Res..

[71] Punjab Energy Development Agency New National Biogas and Organic Manure Management Programme (NNBOMP). https://www.peda.gov.in/nnbomp.

[72] Nasery V., Rao A. (2011). Biogas for Rural Communities. Center for Technology Alternatives for Rural Areas, Indian Institute of Technology Bombay. www.cse.iitb.ac.in/sohoni/pastTDSL/BiogasOptions.pdf.

